# High-volume image-guided injection in the chronic recalcitrant non-insertional patellar tendinopathy: a retrospective case series

**DOI:** 10.1186/s40634-020-00299-7

**Published:** 2020-10-10

**Authors:** Torsten Grønbech Nielsen, Lene Lindberg Miller, Bjarne Mygind-Klavsen, Martin Lind

**Affiliations:** Aarhus University Hospital, Orthopedic Department, Palle Juul-Jensens Boulevard 99, 8200 Aarhus N, Denmark

**Keywords:** High-volume image-guided injection, HVIGI, Patellar tendinopathy, Patella tendon, Eccentric training, Heavy slow resistance training

## Abstract

**Purpose:**

To evaluate if High-volume Image-guided Injection (HVIGI)-treatment for chronic Patellar tendinopathy (PT) improve function and reduce pain at 16-months follow-up.

**Methods:**

Patients with resistant PT who failed to improve after a three-month eccentric loading program were included in the study. Maximal tendon thickness was assessed with ultrasound. All patients were injected with 10 mL of 0.5% Marcaine, 0.5 mL Triamcinolonacetonid (40 mg/mL) and 40 mL of 0.9% NaCl saline solution under real-time ultrasound-guidance and high pressure. All outcome measures were recorded at baseline and at 16 months. A standardised Heavy Slow Resistance rehabilitation protocol was prescribed after HVIGI-treatment. Clinical outcome was assessed with the Victorian Institute of Sports Assessment-Patella tendon questionnaire (VISA-P) and statistically analyses were performed.

**Results:**

The study included 28 single treatment HVIGI procedures in PT in 23 patients (19 men, 4 women) with a mean age of 30.3 (range 19–52). The mean duration of symptoms before HVIGI was 33 months. The baseline VISA-P score of 43 ± 17 (range 15–76) improved to 76 ± 16 (range 42–95) after 16 months (*p* < 0.01). Of the 28 HVIGI procedures 12 patients (15 PT) were not satisfied after the initial HVIGI procedure. Of these, 5 patients (5 PT) had additional HVIGI, 2 patients (2 PT) had corticoid injection and 6 patients (8 PT) needed surgery. Of the remaining 11 patients (13 PT), 9 patients had more than a 13-point improvement in the VISA-P score after 16 months.

**Conclusions:**

In this retrospective case-study, only 9 patients (32%) did benefit of a single HVIGI treatment at 16-months and a 33-point significant improvement was seen on the VISA-P score.

## Background

Jumper’s knee or patellar tendinopathy (PT) is a common overuse injury among athletes. Overall prevalence of PT is seen in 10–14% of elite athletes. The prevalence is higher in sports with high demands on speed and power [13, 16, 20]. Lian et al. presented prevalence as high as 32–45% in elite volleyball and basketball players [20].

In nonelite athletes prevalence of PT is seen in 5.8–8.5% of athletes and again prevalence is higher in speed and power activities such as volleyball and basketball (range 11.3–14.4) [8, 24].

PT is a degenerative condition and could be characterized by a neovascularisation of the patella tendon [15, 18]. The area of degeneration generally occurs distal of the inferior pole of the patella and in the proximal portion of the patella tendon [14].

In 2008, Chan et al. performed the first High-volume Image-guided Injection (HVIGI) study for chronic non-insertional achilles tendinopathy. HVIGI was used in a cohort of patients who failed a three-month guided eccentric training program (ET). The mechanism behind the effect of HVIGI-treatment is believed to be mechanical stretching, breaking or occluding the neovessels and the accompanying nerve ingrowth [9]. This is believed to reduce the pain of the tendinopathy.

Various treatment strategies for PT exist in the literature including eccentric training (ET), platelet rich plasma injections (PRP), shock wave, corticoid injection, load modification, electrolysis etc. These treatments have been performed with various results over the past decades [2, 4–7, 10].

Two systematic reviews and meta-analyses from 2019 investigated non-surgical interventions ability to improve function and reduce pain in PT [4, 10] and concluded that PRP had the greatest improvement and pain reduction compared to the other non-surgical modalities in the long term.

No published HVIGI randomized controlled study (RCT) exists in the literature therefore no studies were included in the meta-analysis from 2019 [10]. A study from 2018 compares HVIGI with PRP and with HVIGI+PRP. Of the three groups HVIGI+PRP is more effective in the short time than the other treatment [3].

Other treatment options on PT are presented in a consensus from ESSKA Basic Science Commitee (2018), which includes: ultrasound guided galvanic electrolysis technique (USGET), mesenchymal stem cells, gene therapy, biomaterials and surgical approach [1]. These treatments will not be presented further in this present study.

The aim of the current study was to evaluate if HVIGI-treatment for chronic PT improve function and reduce pain at 16-months follow-up.

It was hypothesised that HVIGI would result in clinically relevant improvements of symptoms and function for chronic Patellar tendinopathy after 16 months and that most of the patients would benefit from HVIGI treatment.

## Methods

In this retrospective case series of 28 single HVIGI procedures followed prospectively were performed between May 2013 and October 2016. Patients were included if they had a diagnosis of tendinopathy (here defined by (1) pain localized to the inferior pole of patella and (2) neovascularisation and innervation in and around the tendon using ultrasound examination) and had failed a three-month Heavy Slow Resistance training (HSR) program [19]. Patients were excluded if they have had prior HVIGI treatment, patella surgery or were lost to follow up.

The Local Ethics Committee was contacted and determined that an approval for this study was not required (1–10–72-1-19). All data was managed with strict confidentiality and data has been anonymised prior to analysis. Three skilled orthopaedic surgeons diagnosed the patients on the basis of their medical history, physical examination and ultrasound evaluation.

### Intervention

#### Injection

Patients were placed in the supine position with knee in full extension. The needle was inserted in the interface between the Hoffa’s fat pad and the patella tendon. PT injection was performed with 10 mL of 0.5% Marcaine, 0.5 mL Triamcinolonacetonid (40 mg/mL) and 40 mL of 0.9% NaCl saline solution under real-time ultrasound guidance aiming at the area of maximal neovascularisation.

#### Rehabilitation

In the first 72 h after the injection, patients were only allowed to participate in the normal activities of daily living. Running, jumping and heavy resistance training was prohibited. After 72 h, patients were allowed to begin HSR as described by Kongsgaard [19]. A two-page leaflet with exercises and guidelines was given to the patient. Patients knew the rehabilitation due to a previously failed three-month rehabilitation program. If patients had the intention of returning to running and were pain-free, they were educated in a graded running program starting 1 month after HVIGI treatment.

### Clinical outcome measure

The Victorian Institute of Sports Assessment-Patella tendon questionnaire (VISA-P) [23] was used as a primary outcome measure and was recorded at baseline and 16 months. This score ranged from 0 to 100, where 0 was worst and 100 was asymptomatic. Primary outcome was the delta value between baseline and the 16-months follow-up.

A successful outcome was defined as single HVIGI treatment, no subsequent other injection or conversion to surgery and a 13-points improvement [17] at the VISA-P at the 16-months follow-up.

### Ultrasound evaluation

Maximal tendon thickness and neovascularisation were assessed with ultrasound and power Doppler in the supine position with full knee extension. The Patella tendon was scanned in both transverse and longitudinal planes. Ultrasound evaluation was performed at baseline and 16-months follow-up.

### Statistical analysis

Descriptive statistics were calculated. Continuous data were presented as mean ± standard deviation (SD). Categorical data were presented as quantity and proportions (%). Normality was tested by qq-plots. All data were normally distributed, and the student’s T-test was used to compare differences between time-points. Minimal clinical important difference (MCID) was set at 13 points [17]. *P*-values below 0.05 were considered significant. All data was analysed in MS Excel 2010 version 14.0.7237.5000 (32-bit) and STATA 16.1 software (StataCorp, College Station, TX, USA).

## Results

**Fig. 1:**
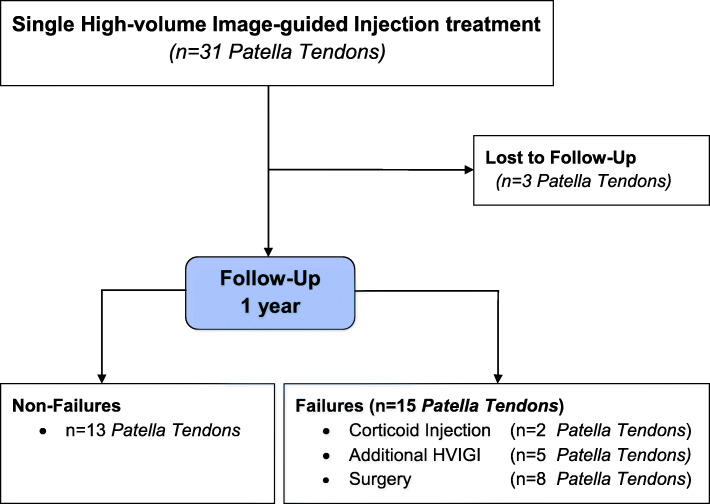
Flow Chart

Thirty-one single treatment HVIGI procedures were performed in the period 2013–2016. The study included 28 PT in 23 patients (19 men, 4 women) (Fig. 1).

The mean age was 30.3 years (range 19–52). The mean duration of symptoms before HVIGI was 33 months (range 12–108).

Patient characteristics are listed in Table 1. Some patients had tried several other conservative treatments apart from HSR before HVIGI including corticosteroid injection or shock wave (Table 1).

**Table 1:** Patients demography

HVIGI procedures/patients, n	28/23
Men/Women, %	83/17
Mean age in years (range)	30.3 (19–52)
Mean pain duration in months (range)	33 (12–108)
Intervention before HVIGI	
● → Corticosteroid Injections, n (%)	17 (61%)
● → Shock Wave therapy, n (%)	1 (4%)

The baseline VISA-P score of 43 ± 17 (range 15–76) improved significantly to 76 ± 16 (range 42–95) at the 16 months follow-up (*p* < 0.01). VISA-P scores are listed in Table 2. Nine patients (32%) having a single HVIGI treatment had above 13-point improvement.

**Table 2:** VISA-P and Patella tendon thickness

	Baseline	16 Months	***p-value***
Patella Tendons/Patients	28 / 23	13 / 11	
VISA-P completeness, n (%)	28 (100)	13 (100)	
VISA-P, mean	43 ± 17	76 ± 16^a^	*< 0.01*
> 13 points improvement, n (%)		9 (32)	
Patella tendon thickness, mm	7.1 ± 1.3	6.1 ± 2.0	*0.10*

Results are presented as mean-values with standard deviation (±SD). ^a^ significant improvement from baseline

The thickness of the Patella tendon measured at baseline was 7.1 ± 1.3 mm compared to 6.1 ± 2.0 mm at the 16 months follow-up (*p* = 0.10) (Table 2).

Of the 31 PT treated for a single treatment with HVIGI 3 patients (3 PT (10%)) were lost to follow up.

Of the remaining 28 PT (23 patients), 12 patients (15 PT (54%)) were not satisfied after a single HVIGI procedure and required further treatment. Of these, 5 patients (5 PT) needed additional HVIGI, 2 patients (2 PT) needed corticoid injection and 6 patients (8 PT) needed surgery.

No complications in relation to HVIGI treatment were observed.

## Discussion

The primary finding of the present study was that a successful outcome after a single HVIGI treatment in patients with PT who had previously failed exercise based treatment was seen in 32% of patients after 16 months. A significant improvement was demonstrated compared to the baseline VISA-P score. The improvement for successful treatments in the VISA-P score from baseline to the 16 months follow-up was 33 points.

Crisp, Morton, Maffulli and Abate found an improvement from baseline to 3–15 months in their studies at 22, 18, 29 and 10 points, respectively [3, 12, 21, 22].

The study from Crisp in 2008 was the first study presenting data from HVIGI procedures in PT [12]. Morton and Maffulli used the same cohort as Crisp (9 patients) in their respective studies from 2014 and 2016 [21, 22]. Differences between these 3 studies are the follow up time point varying from 3 to 15 months and VISA-P at the follow up time point. A tendency in these studies was that longer follow up period lead to better outcome.

Maffulli found a 29 points improvement at 15 months follow up, which was comparable with the present study, but patients were allowed to have more than one HVIGI treatment if first HVIGI treatment was insufficient. Twenty-five of the 44 patients (56%) in their cohort had more than one HVIGI treatment and 3 patients were scheduled for surgery [21].

A difference between these studies and the present study could be the long duration of symptoms in the present patient cohort. Thirty-three months was the time between onset of Patella symptoms and HVIGI procedure compared to 3–21 months in the above-mentioned studies. Except for Abate’s study, the 3 other studies used the HVIGI to recalcitrant PT with duration of symptoms of more than 18 months before HVIGI treatment.

In Abate’s study, patients had not failed a three-month ET/HSR or tried other interventions. HVIGI was used as primary treatment and was performed twice with 14 days apart.

No difference between injection techniques and rehabilitation regimens was observed in the studies listed in Table 3.

**Table 3:** Studies presenting data on High-volume Image-guided Injection (HVIGI)

	Pts (n)	Age (y)	DoS (mt)	Post (mt)	US Δ (mm)	VISA-P pre	VISA-P post	VISA-P Δ	ET/HSR failed
**Crisp** [12] **(2008)**	9	29	21	9	/	46	68	22	YES
**Morton** [22] **(2014)**	20	31	20	3	1.0	46	64	18	YES
**Maffulli** [21] **(2016)**	44	35	18	15	1.2	46	75	29	YES
**Abate** [3] **(2018)**	30	37	3	6	/	53	63	10	NO

VISA-P is presented as mean-values with standard deviation (±SD). Pts = patients, mt = months, DoS = Duration of symptoms, Post = Latest follow-up, US = tendon thickness between baseline and Latest follow-up, Δ = improvement from pre to post, ET/HSR failed = Eccentric Traning/HSR failed before HVIGI, /=unknown

Combining this issue of a high proportion of our cohort having previous corticosteroid treatment with the long duration of symptoms of mean 37 months, indicates that the HVIGI treatment in the present study served as a salvage procedure for PT when other treatments had failed. This could explain the low treatment response rate compared to previous studies in patients with less chronic conditions.

In the present study, a successful outcome was defined as one single HVIGI treatment, no subsequent conversion to surgery and a clinically relevant 13 point improvement at the VISA-P at the 16-months follow-up.

A successful outcome by Abate was a VISA-P improvement above 20 points from baseline to 6-months follow-up. Only 2 patients (11%) had a successful outcome which is one third the success rate of the present study in which a > 20 points improvement was seen in 9 patients (32%).

Time from baseline to follow-up might be an important factor in the treatment of PT.

In the present study, a reduction of 1.0 mm in Patella tendon thickness after HVIGI treatment after 16 months was found. Other HVIGI studies have found reduced thickness of 1.0 to 1.2 mm at 3 and 15 months, respectively [21, 22].

It appears, based on the present study, that patients with long-lasting PT who have failed a three-month ET, corticosteroid injection or shockwave will have a 32% chance of a clinically relevant improvement in subjective outcome after a single HVIGI treatment after 16 months. These results indicate that patients with chronic PT and failed previous treatment should be counselled about the limited success rate of further treatment with HVIGI. This limited success rate could be explained by the pathomechanics in which patella tendon overload results in collagen degeneration, collagen disorganization and neovascularisation and innervation both in and around the tendon [11]. The patients have performed a 3 months specific training program before the HVIGI intervention with the intention to normalize the tendon tissue, as described by Kongsgaard [19]. As the patients did not benefit of the HSR training, HVIGI injection was performed with the purpose of peritendinous mechanical stretching, breaking the fibrosis and neovessels and the accompanying nerve ingrowth. HVIGI treatment does not address the intra tendinous pathologic changes and this could be a main factor for why only 32% of the patient had a clinical relevant improvement at 16 months follow-up.

The HVIGI treatment is found safe, due to no complications observed regarding this cohort.

Several limitations were acknowledged in this study. The most important were related to the absence of a control group. Also training compliance is unknown due to the lack of a training dairy. Vascularisation and neovascularisation were not measured at baseline and 16 months. Functional testing such as; gait analysis, strength assessment etc. could be beneficial in the analysis of the outcome after the HVIGI intervention. Another limitation could be NaCl saline solution in combination with Triamcinolonacetonid (cortisone) which could have an influence on the results, despite the dose of Triamcinolonacetonid was only 20 mg per HVIGI procedure.

## Conclusion

In this study 32% of the patients with chronic PT who had failed Heavy slow resistance training treatment did benefit of a single HVIGI treatment. Among the non-failures a significant 33-point improvement in VISA-P score was seen at 16-months follow-up.

## Data Availability

The datasets used and/or analysed during the current study are available from the corresponding author on reasonable request.

## Abbreviations

PT: Patellar Tendinopathy
HVIGI: High-volume Image-guided Injection
ET: Eccentric training
PRP: Platelet Rich Plasma
RCT: Randomized controlled study
HSR: Heavy Slow Resistance Training
VISA-P: Victorian Institute of Sports Assessment-Patella tendon questionnaire
MCID: Minimal clinical important difference
